# The chaperone DNAJB6b halts amyloid formation through association with transient Aβ oligomers

**DOI:** 10.1039/d6cp00678g

**Published:** 2026-04-29

**Authors:** Josef Getachew, Andreas Carlsson, Emil Axell, Dev Thacker, Ulf Olsson, Sara Linse

**Affiliations:** a Biochemistry and Structural Biology, Lund University 22100 Lund Sweden josef.getachew@chem.lu.se; b Physical Chemistry, Lund University 22100 Lund Sweden

## Abstract

Oligomers are transient toxic species in amyloidoses such as Alzheimer's disease. The binding of oligomers by human chaperone proteins has been inferred from the lack of detectable interactions with monomeric amyloid proteins and delay of fibril formation at sub-stoichiometric chaperone to monomer molar ratios. In this study, we provide direct experimental evidence for the binding of the human chaperone DNAJB6b (JB6) to amyloid peptide oligomers formed during an ongoing fibril formation process leading to the stabilization of these transient species. JB6 is a potent inhibitor of the aggregation of multiple amyloid peptides and here we observe the inhibition of the model amyloid-β (Aβ) 20–34 peptide at an astounding sub-stoichiometric 1 : 100 000 ratio of chaperone to amyloid peptide. Through microfluidic diffusional sizing, we detect an increase in the average hydrodynamic radius of JB6 when added to the supernatant of samples withdrawn from an ongoing fibril formation process, implying an interaction with transient non-monomeric Aβ20–34 and Aβ42 species, which we interpret as oligomers. Furthermore, the oligomer stability towards dissociation was studied using the same method. The results imply that JB6 stabilizes the oligomers against dissociation.

## Abstract 2

Inhibition of amyloid-β fibril formation by the human chaperone DNAJB6b through the binding of transient oligomers, detected with microfluidic diffusional sizing.

## Graphical abstract

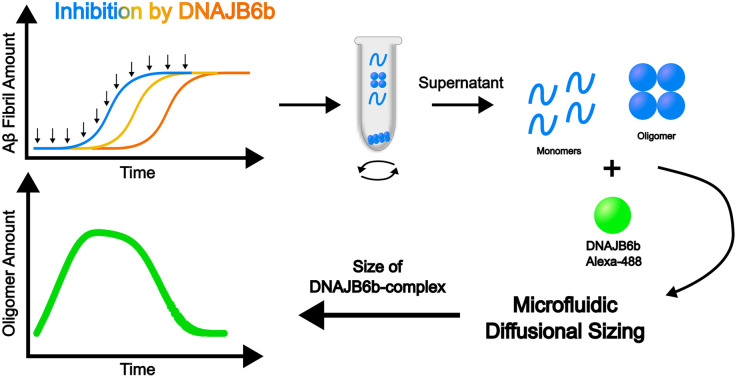

## Introduction

The misfolding, aggregation, and fibril formation of amyloid proteins are involved in several neurodegenerative diseases, including Alzheimer's and Parkinson's disease. Each disease is linked to one or several amyloid proteins or peptides. In the case of Alzheimer's disease, amyloid-β peptides 1–40 and 1–42 (Aβ40, Aβ42) and several isoforms of the protein tau are implicated.^1–7^

During fibril formation, various transient amyloid species coexist, referred to as oligomers, which are intermediates in primary and secondary nucleation pathways.^8^ Transient in nature, low in abundance, and representing only a small fraction of the total protein population with more abundant end state monomers and fibrils, oligomers are particularly challenging to study. Their stability and time-dependent concentration vary with the type of protein and solution conditions, further adding to the complexity, as reviewed.^9,10^ Oligomers may form in solutions supersaturated with protein monomers, and their rate of dissociation is typically higher than the rate of structural conversion to form the nuclei of more stable amyloid fibrils.^11–15^ Several *in vitro* and *in vivo* studies have demonstrated that Aβ oligomers are toxic, suggesting that these oligomers represent a prime target for therapeutic intervention.^16–18^

A native defense against protein misfolding and aggregation is provided by a group of proteins called chaperones.^19,20^ DNAJB6b (JB6) is a class B chaperone protein of the DNAJ family (previously Hsp40), which are known to increase the affinity of the chaperone Hsp-70 to misfolded/aggregated proteins (clients), *via* their J-domain.^21,22^ Together with Hsp110, they prevent protein aggregation or facilitate disaggregation^23^ and degradation^24,25^ of aggregates. However, JB6 has also been reported to prevent *in vitro* aggregation of Aβ42,^26,27^ Aβ40,^28^ PolyQ peptides^22,29–31^ and α-synuclein,^32^ independently of Hsp70.

Chaperone-client interactions, leading to retardation of amyloid formation, are expected to be short range, and involve co-assembly of chaperone and client.^33^ JB6 retards amyloid formation even at very low stoichiometric [JB6]/[client] ratios.^26,28,30,32^ This cannot be reconciled by binding of a small fraction of client monomers to JB6, since this would only lead to a very small retardation at low JB6 concentrations. Monomer binding was not experimentally detectable in the case of JB6 and α-synuclein.^32^ The low stoichiometric ratio required for retardation thus suggests that the underlying interaction involves low abundance species, such as oligomers.^26,32^ Previous work using mass-spectrometry (MS) detected a decrease in the population of small ionizable oligomers of Aβ40 after incubation with JB6.^28^

**Fig. 1:**
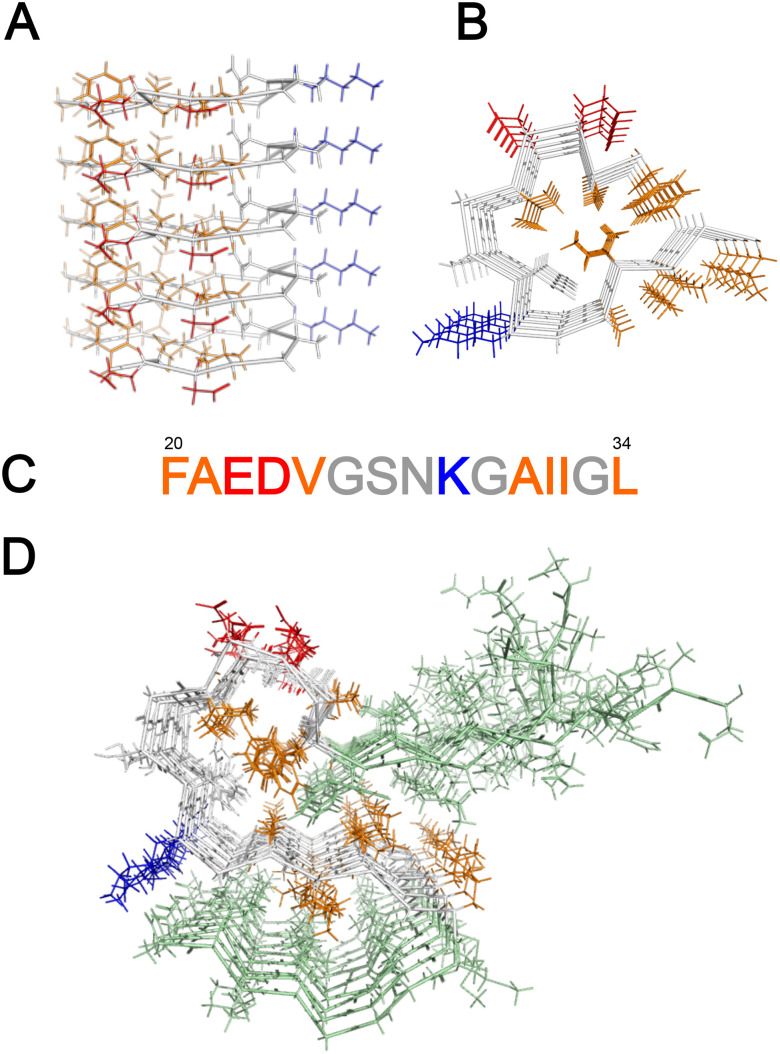
Sequence and structure of the Aβ20–34 and Aβ42 fold in fibrils, adapted from PDB: 6OIZ and 5KK3, respectively. In orange are hydrophobic side chains, in blue and red are positively and negatively charged side chains, respectively. (A) Five Aβ20–34 monomer planes in a protofilament with 4.8 Å stacking distance, viewed perpendicular to the fibril axis. (B) The monomer fold of Aβ20–34 viewed along the fibril axis. Most of the hydrophobic residues are buried in the core of the fibril whereas charged residues are pointing outwards. The side-chains of I32 and L34 appear on the surface of the monomer but are in the fibril buried in an interface with another monomer.^35^ (C) Aβ20–34 sequence. (D) The monomer fold of Aβ42 viewed along the fibril axis. In green are residues 1–19 and 35–42. In the Aβ42 fibril, the side-chain of L34 is not solvent exposed, but buried in the interface with another monomer.^37^

**Fig. 2:**
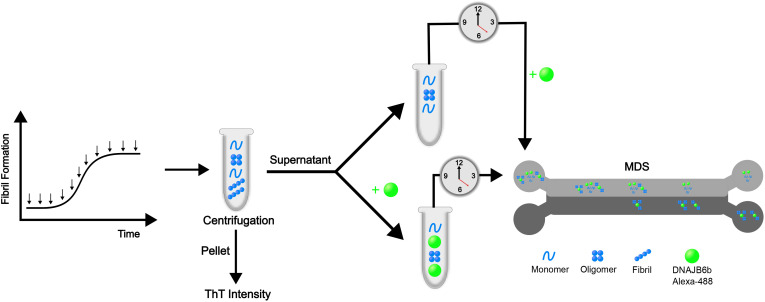
Schematic illustration of the experimental setup. Non-seeded samples collected at different timepoints during incubation (37 °C, with 1000 RPM stirring) were centrifuged for 30 minutes at 14 100 RCF. The supernatant was collected and mixed with Alexa-488-JB6 and studied by MDS to obtain the average hydrodynamic radius of the fluorescent species. MDS utilizes the diffusivity of differently sized molecules by measuring the fluorescence intensity in two chambers at the end of a capillary and from that calculate the average hydrodynamic radius. By incubating samples with or without JB6, and measuring over time, the stability is followed. For samples incubated without JB6, the labelled chaperone is added just prior to measuring. The protein and peptide species in this illustration are not in scale.

In the current work, we employ microfluidic diffusional sizing (MDS) to detect oligomers in solution during an ongoing aggregation process.^34^ Using this method, we investigate the co-assembly of JB6 with oligomers of Aβ20–34, which is a 15-residue peptide fragment (Fig. 1A–C) of the amyloid precursor protein (APP). As depicted in Fig. 2, we may thus detect any species interacting with JB6 as an increase in size. This approach was also applied to JB6 and Aβ42 to show its viability in a physiologically relevant system.

Proteolysis of APP generates different alloforms of the Aβ peptide, of which Aβ40 is most prevalent and Aβ42 most closely associated with Alzheimer's disease. The choice of the model peptide Aβ20–34 is motivated by its relatively high aqueous solubility, allowing for experiments at higher concentrations (millimolar) compared to the nanomolar to micromolar range normally used in studies of amyloid proteins.^35,36^ The structure of its ordered solid state is known (Fig. 1A–C);^35^ the peptide folds in two dimensions, and stacks with intermolecular β-sheets parallel to the fibril axis and perpendicular to the folding plane, analogous to *e.g.* Aβ42 fibrils (Fig. 1D).^37^ Complementary techniques such as cryogenic transmission electron microscopy (cryo-TEM), small angle X-ray scattering (SAXS) and thioflavin-T (ThT) fluorescence spectroscopy were employed to examine the fibril assembly.

## Results and discussion

### Inhibition at low sub-stoichiometric molar ratios

The inhibition of amyloid fibril formation by JB6 at sub-stoichiometric levels has been reported for multiple pathological clients. Common to these studies, the concentration of amyloid client is in the 1–100 µM range and JB6 down to single digits nanomolar, giving a molar ratio range of 1:100–1:10 000.^26,28,30,32,38,39^

**Fig. 3:**
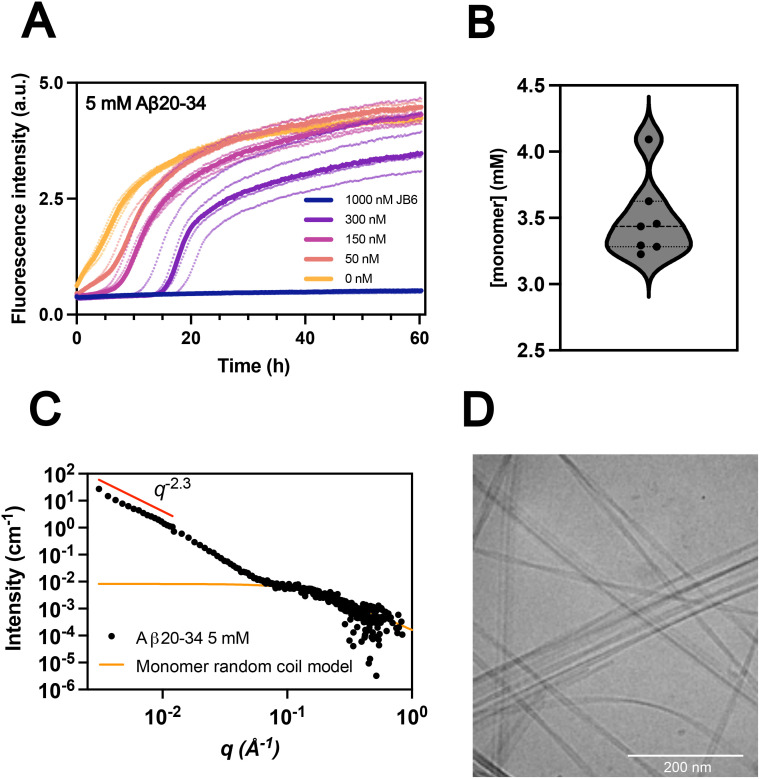
Seeded Aβ20–34 aggregation (A) aggregation kinetics of 5 mM Aβ20–34 supplemented with 0.7% seeds at time zero, at 37 °C and pH 7.4, in the absence and presence of 50–1000 nM JB6. ThT fluorescence intensity was used to monitor the aggregation process (full kinetic traces available in Fig. S1). The medians of *N* = 5 replicates for 50–1000 nM JB6, and *N* = 7 replicates for 0 nM JB6, are plotted as solid lines, with individual traces as dots. (B) Aβ20–34 solubility measured at the end of the aggregation process by absorbance in HPLC, yielding a mean solubility of 3.5 ± 0.3 mM (standard deviation, STD), from *N* = 7 measurements. (C) and (D) Characterization of Aβ20–34 fibrils in the absence of JB6. (C) The SAXS pattern of a sample after aggregation (Fig. S1), 6 days. Fibril scattering dominates at low *q*, while monomer scattering dominates at higher *q*-values. Monomeric Aβ20–34 is modelled as a random coil with a concentration of 3.9 mM obtained from the scattered intensity. (D) Cryo-TEM image of a 5-fold diluted fibril sample taken after 6 days of incubation, *i.e.* at the plateau of the aggregation process (Fig. S1). Additional images are shown in Fig. S5. Cryo-TEM and SAXS characterization of samples in the presence of JB6 are shown in Fig. S6.

Here, the aggregation of 5 mM Aβ20–34 was studied at 37 °C, pH 7.4 in solutions supplemented at time zero with 35 µM seeds (0.7%, monomer equivalents) and 0–1 µM JB6. The aggregation was monitored by recording the fluorescence intensity of the dye thioflavin-T (ThT), which is known to bind to amyloid fibrils with enhanced quantum yield. The results are presented in Fig. 3A. As can be seen, the aggregation is significantly retarded at remarkably low JB6 concentrations with 50 nM JB6 leading to almost a doubling of the half time of aggregation, *t*_1/2_ (Fig. 3A, Fig. S1 for full kinetics). This corresponds to a molar ratio of 1 : 100 000 chaperone to Aβ20–34, which is, to our knowledge, the lowest reported sub-stoichiometric ratio causing an inhibition of an amyloid client by JB6. Interestingly, the shapes of the kinetic traces beyond the lag phase are very similar. Thus, the main chaperone effect seems to be an increase of the effective lag time with increasing JB6 concentration. Attempts to fit the kinetic traces using master equations covering the mechanistic steps in amyloid formation^40^ are discussed in Section S2. In summary, it was not possible to fit the data in the presence of JB6 by assuming changes in either *k*_+_ or *k*_2_. Instead the data are well fitted under the assumption that the seeds are made inactive by the presence of JB6.

The most active species of JB6 are the monomeric subunits,^41,42^ which are the dominating species in solution up to the critical micelle concentration, cmc ≈120 nM,^43^ and above this concentration they are present at roughly the cmc concentration in co-existence with micelles. We observe a retardation of Aβ20–34 aggregation at JB6 concentrations below the cmc, as well as above. Above the cmc, JB6 micelles may act as a reservoir of monomers because the dissociation rate of JB6 micelles is high compared to the Aβ20–34 aggregation rate; while *t*_1/2_ without JB6 is ≈6–7 h, dissociation of JB6 micelles at 37 °C displays a half-time of ≈20 min.^42^

### Aβ20–34 solubility

To confirm previous findings of a high solubility of Aβ20–34^36^ at 37 °C and pH 7.4, the peptide concentration in the supernatant was analyzed using HPLC with absorbance at 256 nm after pelleting fibrils at the end of the aggregation reaction. The result of *N* = 7 individual measurements are presented in Fig. 3B. We conclude that the solubility is 3.5 ± 0.3 mM, which is on the order of 10 000 and 100 000 times higher than reported for Aβ40 and Aβ42, respectively.^44–46^ As a second approach, the monomer concentration was followed using nuclear magnetic resonance (NMR) spectroscopy (Fig. S3).

### Characterization of Aβ20–34 fibrils

Next, we characterized the aggregates formed in the absence of JB6 to determine whether the ThT positive Aβ20–34 aggregates formed in our system are amyloid fibrils. The cryo-TEM images (Fig. 3D and Fig. S5) show the presence of long fibrils with a cross-section dimension on the order of 10 nm, with some variation. Under the current solution conditions, the SAXS pattern at low *q*-values displays a steep power law dependence with *I*(*q*) proportional to *q*^−2.3^ (Fig. 3C), while *q*^−1^ would be expected for a fibrillar structure. This is indicative of attractive fibril–fibril interactions and a heterogeneous fibrillar network.^47,48^ At higher *q*-values, >0.06 Å^−1^, the scattering is dominated by the peptide monomers. By modelling monomeric Aβ20–34 as a random coil, its contribution to the scattered intensity was estimated as described in Section S6. A model scattering curve was calculated using the SasView software^49^ and adjusted to give a good description of high *q*-data (yellow line in Fig. 3C). From the scattered intensity we estimate the monomer concentration, coexisting with fibrils, to be 3.9 mM, in good agreement with the solubility measurements using HPLC (Fig. 3B). We estimate a radius of gyration of the monomer random coil to be 1 nm, from the scattering at higher *q*-values (*q* > 0.1 Å^−1^). Cryo-TEM and SAXS characterization of samples in the presence of JB6 were also conducted (Fig. S6). No differences were observed, possibly due to the low stoichiometric ratios not being sufficient for observable changes in the ultrastructure.

### Association of JB6 with transient Aβ20–34 species

To investigate the possible association of JB6 with Aβ20–34 oligomers, we performed an experiment in which samples were withdrawn during an ongoing aggregation process of 6.5 mM Aβ20–34 at 37 °C, pH 7.4, with stirring. Samples were centrifuged to sediment the fibrils, and Alexa-488-JB6 was added to the supernatant, providing a solution with 12.5 nM JB6 and 75% (v/v) supernatant. After 5 minutes of incubation, the average hydrodynamic radius, 〈*R*_h_〉, was measured using MDS. An apparent increase of the chaperone 〈*R*_h_〉 reflects on association of JB6 with Aβ20–34 species in the supernatant.

**Fig. 4:**
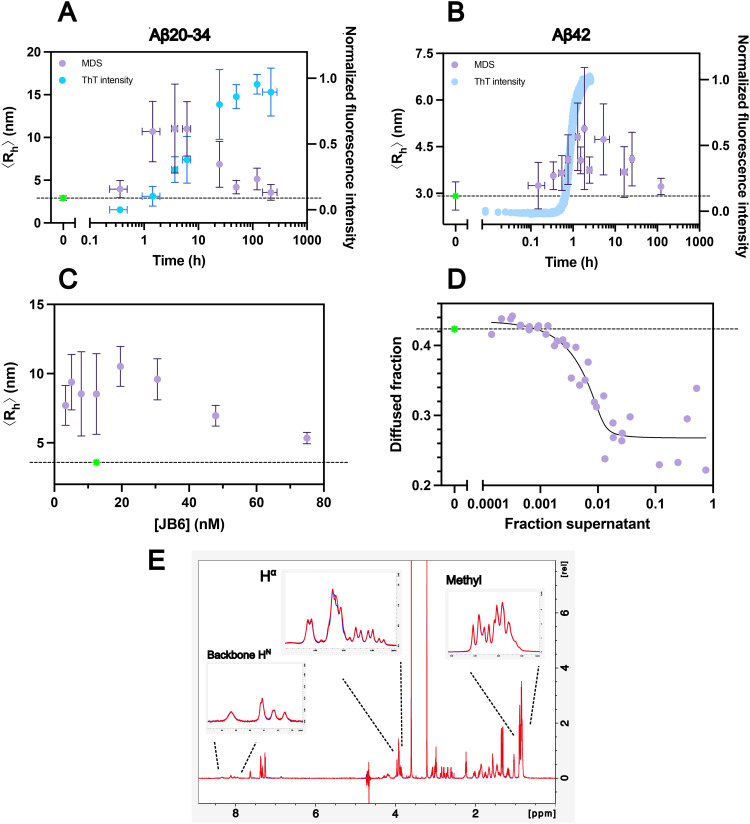
Detection of JB6 binding to Aβ20–34 and Aβ42 oligomers. Alexa-488-labelled JB6 is mixed with the supernatant of sedimented samples withdrawn from an ongoing aggregation process and the average hydrodynamic radius is measured using MDS (Fig. 2). The 〈*R*_h_〉 of JB6 mixed with monomeric amyloid peptide, which gives approximately the same value as for JB6 alone (≈3 nm) is shown in green, while all other 〈*R*_h_〉 values are shown in purple. (A) The 〈*R*_h_〉 of JB6 (12.5 nM final concentration) mixed with supernatant *versus* time of withdrawal from the Aβ20–34 aggregation process. The data are pooled from 3 repeat experiments, with MDS data plotted as mean with *y*-axis error bars representing the STD from 7 ≤ *N* ≤ 15 replicates. In blue is shown the ThT fluorescence intensity of the centrifuged sample after re-mixing of supernatant and pellet, subsequent to the MDS measurements. ThT data are plotted as mean values with error bars representing the STD from *N* = 3 or 4 replicates. Points without error bars are from *N* = 2 replicates. (B) The 〈*R*_h_〉 of JB6 (12.5 nM final concentration) mixed with supernatant *versus* the time of Aβ42 aggregation process. The data are pooled from 4 repeat experiments, with MDS data plotted as mean with *y*-axis error bars representing the STD from 7 ≤ *N* ≤ 9 replicates. The *x*-axis error bars are STD from 3 ≤ *N* ≤ 5 replicates, points without error bars are from *N* = 2 datasets. In blue is shown the ThT fluorescence intensity, measured continuously in a plate reader from one dataset with *N* = 3 replicates. (C) The 〈*R*_h_〉 of JB6 (3–75 nM) titrated to samples withdrawn from an ongoing Aβ20–34 aggregation process, diluted to correspond to a supernatant fraction of 2.5% (v/v). Data are pooled from two separate aggregation processes, with *y*-axis error bars from STD of 4 ≤ *N* ≤ 6 replicates. (D) The diffused fraction of JB6 mixed with supernatant of samples withdrawn from an ongoing Aβ20–34 aggregation process. The supernatant was present at a fraction of 0.014–75% (v/v) while the JB6 concentration was constant at 12.5 nM. Data are pooled from two separate aggregation processes, where the supernatant is extracted from centrifuged samples taken at the broad peak between 2 h and 7 h. The data are fitted using an equation for independent binding (eqn (1) in the Methods section). (E) Superimposed 1D ^1^H NMR spectra recorded for 100 µM Aβ20–34 with (red) or without (blue) 10 µM JB6. The backbone H^*N*^, H^*α*^ and methyl regions are enlarged.

We find that the 〈*R*_h_〉 of JB6 incubated with monomeric Aβ20–34 remains around ≈3 nm (Fig. 4A), which is the hydrodynamic radius of the JB6 monomer.^42^ As the aggregation process progresses, there is an increase in 〈*R*_h_〉, beginning already in the lag phase of the Aβ20–34 aggregation, with a peak at around *t*_1/2_ (Fig. 4A). At the peak, 〈*R*_h_〉 ≈11 nm, but eventually, the size returns to the initial value of chaperone alone around 3 nm. These data imply that JB6 interacts with transient amyloid oligomers (attempts to fit the oligomer population are shown in Section S7). In this work, we use an operational definition of Aβ20–34 oligomers as any multimers remaining in solution after centrifugation at 14 100 RCF for 30 min, which are large enough to give a measurable change in JB6's 〈*R*_h_〉, and are small enough to flow in the MDS capillary (a control experiment in Sections S8 and S9 shows that mature fibrils do not flow in the MDS capillary).

### Low affinity of JB6 for Aβ20–34 monomers

Since an association between monomeric Aβ20–34 and JB6 would not change the 〈*R*_h_〉 beyond the standard deviation of the measurements, it is not detectable by the above described MDS experiments. Still, we can infer that the affinity is significantly higher for oligomers than for monomers, as the oligomers are likely present at a much lower concentration than the monomers, but still outcompete any monomer binding. However, to directly examine this interaction, we recorded one-dimensional (^1^H) NMR spectra of 100 µM Aβ20–34 with or without 10 µM JB6 (10%) (Fig. 4E). In these experiments, the Aβ20–34 concentration was below the solubility and the peptide should therefore be fully monomeric. The absence of major changes in chemical shifts or intensities under these conditions, illustrated by superimposed spectra, supports the interpretation that there is no strong binding between monomeric Aβ20–34 and JB6. In the H^*α*^ region there is a minor change in intensity and chemical shift, probably arising from one of the three glycines due to strong coupling, meaning that the two protons in at least one glycine are no longer equal.

### Average co-oligomer size

Of importance to our interpretation, the current MDS approach measures the average hydrodynamic radius. If not all JB6 is associated with Aβ20–34 oligomers, the free JB6 monomers (〈*R*_h_〉 ≈3 nm) will contribute to the average, resulting in an underestimate of the average size of any co-oligomers. If instead all JB6 is associated with oligomers, we can interpret the measured size as the actual average hydrodynamic radius of the co-oligomers. To determine whether all JB6 is bound, we titrated JB6 to a constant fraction, 2.5% (v/v), of the supernatant (Fig. 4C). At very low chaperone concentrations, essentially all JB6 will be bound in the case of high affinity, and the MDS will report on the average size of co-oligomers. As the concentration of JB6 increases towards and above saturation, the reported size is an average of co-oligomers and free JB6, and so decreases. From these data, it appears that the JB6 concentration used in the MDS time course experiments, 12.5 nM, is in the regime where all JB6 is bound, and the measured average hydrodynamic radius reflects the average co-oligomer size.

Importantly, the oligomers are captured during an ongoing aggregation process. With an average 〈*R*_h_〉 around 10 nm for the co-oligomers, we can conclude that these cannot consist of just a few Aβ20–34 molecules and one JB6, but are significantly larger aggregates. Previously, small Aβ40 oligomers up to tetramers were found to be captured by JB6.^28^ With our microfluidic approach we are able to detect binding to larger oligomers that may not be ionizable with mass-spectrometry. Together, the previous^28^ and current data imply that JB6 may interact with both small and large oligomers of Aβ.

### Titration of JB6 with Aβ20–34 oligomers

Next, we recorded a binding curve of Aβ20–34 oligomers titrated to 12.5 nM Alexa-488-JB6 (Fig. 4D) by measuring the size of JB6 as a function of supernatant concentration, given as the volume fraction of supernatant. The model is fitted to the raw data, *i.e.*, “diffused fraction” (defined as the fraction of the total fluorescence intensity in the chamber opposite to the sample flow inlet), which is a linear combination of the diffused fractions of free and bound JB6. At low volume fractions of supernatant, the sample is dominated by free JB6, providing an average hydrodynamic radius of ≈3 nm and a high diffused fraction of ≈0.42. As the volume fraction of supernatant increases, the diffused fraction decreases, since the hydrodynamic radius increases upon JB6 binding to oligomers. At high fractions of supernatant, essentially all JB6 is bound and the diffused fraction is constant. The data were fitted (Fig. 4D), assuming independent binding (eqn (1) in the Methods section) yielding a best fit for *K*_D_ at ≈1 nM, signifying a high affinity binding. The error analysis (Section S10), however, shows only a ≈1.25-fold increase in the error square sum (e.s.s.) at *K*_D_ above 100 nM, with no change in e.s.s. at *K*_D_ below 1 nM. Therefore, *K*_D_ can only be concluded to be around 1 nM or lower. Future determination of oligomer concentration or number of binding sites may allow for a more accurate determination of *K*_D_.

### Association of JB6 with transient Aβ42 species

In a complementary experiment, the association between Aβ42 oligomers and JB6 was studied (Fig. 4B) to investigate whether oligomer binding applies to the disease related peptide also. Samples were withdrawn during an ongoing aggregation process, starting from 4 µM monomeric Aβ42 at 37 °C, under quiescent conditions, and centrifuged at 20 000 RCF for 5 minutes. Alexa-488-JB6 was added to the supernatant and analysed using MDS. Again, no change in size of JB6 (≈3 nm) is observed after mixing with monomeric Aβ42. However, over time, we detect an increase in size, with a peak close to or just after *t*_1/2_, followed by a slow return to 3 nm. We note that observations during the faster process for Aβ42 may be more skewed by the handling and operation time plus running time for MDS. The time-evolution is still similar to that reported from isotope-based oligomer quantification.^14^ The peak 〈*R*_h_〉 of ≈5 nm is smaller than found with Aβ20–34, but similar to the S100G-based binding protein for Aβ42 oligomers.^34^ From these results, we can infer that JB6 association with transient oligomers is not specific to the smaller Aβ20–34 fragment but could describe the mechanism of action of JB6 also for Aβ42.

### Aβ20–34 oligomer stability

**Fig. 5:**
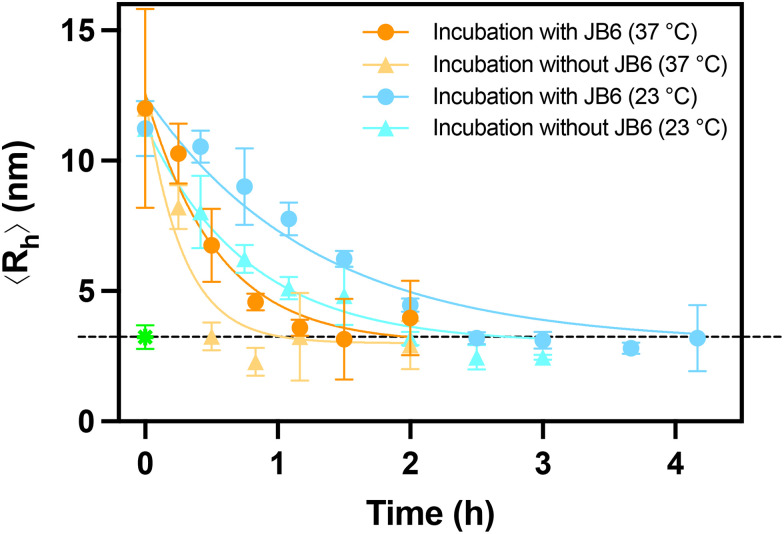
Oligomer size *versus* incubation time at 37 °C and 23 °C. The 〈*R*_h_〉 of Alexa-488-JB6 was measured using MDS for Aβ20–34 supernatant samples to which JB6 was added before (circles) or after (triangles) the incubation period. The green data point is the 〈*R*_h_〉 of JB6 mixed with monomeric Aβ20–34. Data points are plotted as mean with error bars as STD, from *N* = 3 or 4 replicates. The solid lines are fits to the data using a single exponential decay, *a*·e^(−*k*_off_·*x*)^ + *c*.

Having established that JB6 interacts with the transient oligomers formed during an ongoing aggregation process, we can now use the same method to study the stability of the oligomers. While a small fraction of oligomers nucleate to form the more stable fibril assembly, in a number of systems most oligomers dissociate.^9,14^ To determine the stability of Aβ20–34 oligomers, we measured the average hydrodynamic radius over time since their withdrawal. The oligomer-containing supernatants of samples from an Aβ20–34 aggregation process were incubated for various times, after which Alexa-488-JB6 was added and the mixture was analysed with MDS (as illustrated in Fig. 2). This allows us to follow the size of oligomers as a function of time, since JB6 binds in and act as a probe. As can be seen, the ≈10 nm oligomers dissociate with time, implying that they are sub-critical and thus unstable (Fig. 5) in line with earlier observations in other amyloid forming systems.^9,14^ A key outcome of the MDS measurements is the identification of rather large but yet sub-critical Aβ20–34 oligomers, implying that the oligomers reach high aggregation numbers before they convert to a fibrillar structure, as previously observed.^34,50–54^

### JB6 effect on oligomer dissociation rate

The next question of interest is whether JB6 stabilizes the Aβ20–34 oligomers, *i.e.* increases their lifetime against dissociation. This we investigate by incubating the oligomer-containing supernatant in the presence of Alexa-488-JB6 (and compare to the above experiment where the incubation was in absence of JB6) and measure the average hydrodynamic radius as a function of time. We observe that oligomers incubated in the absence of JB6 dissociate approximately twice as fast as those incubated with JB6 (Fig. 5). We estimate *k*_off_ = 3.5 h^−1^ at 37 °C for oligomers incubated in the absence of JB6, and *k*_off_ = 1.9 h^−1^ in the presence of JB6. This indicates that JB6 has a stabilizing effect on Aβ20–34 oligomers. It may be noted that the stability of the oligomers is dependent on the monomer concentration, as the net dissociation rate is higher with less monomers.

As expected for a thermally activated dissociation process, we find that the oligomers dissociate more slowly at room temperature compared to at 37 °C (Fig. 5). At both temperatures JB6 is found to stabilize the oligomers against dissociation, with *k*_off_ at room temperature decreasing from 1.3 h^−1^ for incubation in the absence of JB6 to 0.8 h^−1^ in the presence of JB6.

### Aβ20–34 oligomer dissociation *versus* fibril nucleation

Fibril formation is the spontaneous process and inevitable in a supersaturated solution, where the total peptide concentration is above the solubility of the fibrils. While we detect oligomer dissociation back into monomers, a small fraction of the oligomers will convert into fibrils that grow very fast by monomer elongation and quickly reach a size where they do not enter the MDS channel. If the timescale for fibril formation (Fig. 3A) is longer than that of oligomer dissociation (Fig. 5), as is the case here, we would, however, not detect an increase in size at first, especially given that oligomer dissociation is studied under quiescent conditions as opposed to the aggregation prior to sample extraction and centrifugation, where stirring is used. Given enough time, eventually there should be aggregation in the oligomer samples and an increase in size again. Following the intensities measured with MDS, we detect slightly lower intensities for pure oligomers (Fig. S9B), and there is a decrease in intensity with time at 37 °C; however, the intensities are always higher than for JB6 mixed with pure fibrils, where size determination was not possible with MDS. The decrease in intensity at 37 °C could possibly be caused by the formation of a minor fraction of fibrils that get stuck in the microfluidic channels.

### Potential influence on toxicity and therapeutic development

As oligomers are shown to be toxic, it is interesting to discuss the potential consequences of their stabilization against dissociation by JB6. Co-oligomers with JB6 may have reduced toxicity if JB6 binds to and covers their hydrophobic surface, which is involved in membrane permeation by pure oligomers.^55^ The binding of JB6 to oligomers may also promote their biochemical processing into harmless products.^24,25^ Moreover, if JB6 binding retards conversion to fibrils, this reduces the generation of catalytic surface available for secondary nucleation and thereby a major loss in total oligomer concentration because in an uninhibited case a very vast majority of the oligomers are formed through secondary nucleation.^14,56^

Two of the most well-known therapeutic antibodies against Alzheimer's disease, Lecanemab and Aducanumab, have been shown to bind to aggregated forms of Aβ.^57^ In this project, we are able to investigate the interaction between on-pathway oligomers as opposed to oligomers made off-pathway through different protocols. The design of peptides or antibodies that inhibit secondary nucleation processes to limit oligomer production is an emerging strategy that would benefit from further studies of the mechanism behind the ability of JB6 to stabilize oligomers against dissociation.

## Conclusions

In conclusion, we have detected very low sub-stoichiometric inhibition of the amyloid peptide Aβ20–34 by JB6 at a 1 : 100 000 molar ratio of chaperone to client. Furthermore, we measured an association between JB6 and transient oligomeric species based on an increase in the apparent hydrodynamic radius of fluorescent JB6. Similar association, although with smaller oligomer size was also observed for oligomers of the Aβ42 peptide. The increase in apparent hydrodynamic radius of JB6 was also utilized to estimate the affinity of JB6 for these oligomers, their stability against dissociation and the effect of JB6 on this parameter. Aβ20–34 oligomers were found to dissociate on a time scale of one hour, with slower dissociation in the presence of JB6.

## Methods

### Purification of Aβ20–34

The peptide was purchased in synthetic form with free termini from GenScript (Piscataway, New Jersey), lyophilized, 98.2% purity as a chloride salt. To remove the contaminants and any aggregated peptide, the lyophilized powder was dissolved in 2.2 ml 6 M guanidinium hydrochloric acid (GuHCl), 20 mM sodium phosphate (NaP), 0.02 mM ethylenediaminetetraacetic acid (EDTA), pH 8.0 (set with NaOH), and purified using size exclusion chromatography with a fast protein liquid chromatography (FPLC) system (DuoFlow, Hercules, California). The column used was a Superdex peptide 10/300 GL (GE Healthcare, Chicago, Illinois). Prior to injection on the column, the peptide was incubated for one hour in GuHCl. The buffer used during size exclusion was 20 mM NaP, 0.02 mM EDTA, 0.02% sodium azide (NaN_3_), pH 7.4 (adjusted from 7.3 to 7.4 with NaOH). The protein concentration was determined from the integration of the collected peak in the FPLC chromatogram with absorbance recorded at 256 nm and using the extinction coefficient *ε*_256nm_ = 197 M^−1^ cm^−1^.

### Expression and purification of DNAJB6b

Wild-type JB6 and an *N*-Cys mutant (with a Cys residue added just after the initiating Met) were expressed tag-free in *Escherichia coli* (*E. coli*) BL21 DE3 pLysS star in an autoinduction medium^58^ and purified with sonication, passage through ion-exchange resins, ammonium sulfate precipitation, and two size exclusion chromatography (SEC) steps with and without GuHCl, according to a published protocol.^38^ Subsequently, the *N*-Cys mutant was fluorescently labelled with Alexa-Fluor-488 using the maleimide form of the dye, and excess dye was removed by SEC. After this, the JB6 mutant was judged to be 60% labelled based on the absorbance at 490 and 280 nm.

### Expression and purification of Aβ42

Aβ (M1–42) with the sequence MDAEFRHDSGYEVHHQKLVFFAEDVGSNKGAIIGLMVGGVVIA, here called Aβ42, was expressed in *E. coli* BL21 DE3 pLysS star in LB medium with IPTG induction and purified using sonication, centrifugation and a series of ion exchange and size exclusion steps, as described.^59^ Purified monomeric Aβ42 was aliquoted, lyophilized and stored frozen. Prior to the beginning of each experiment, aliquots were dissolved in 1 ml 6 M GuHCl and once more purified with SEC on a Superdex 75 10/300 Increase GL column using FPLC to again isolate the monomer. The elution buffer was 20 mM NaP, 0.2 mM EDTA, pH 8.0. The eluted peptide was kept on ice to prevent aggregation. The protein concentration was determined from the chromatogram with absorbance recorded at 280 nm using the extinction coefficient *ε*_280nm_ = 1490 M^−1^ cm^−1^.

### Aggregation of Aβ20–34 with and without JB6

The purified peptide was diluted to 5 mM in 20 mM NaP, 0.02 mM EDTA, 0.02% NaN_3_, pH 7.4 and supplemented with 35 µM seeds (0.7%) (sonicated for 2 minutes in a sonication bath), 20 µM ThT, and JB6 in concentrations of 0 nM, 50 nM, 150 nM, 300 nM or 1000 nM. Each solution was added to at least 5 wells of a Corning 96-well clear bottom half area PEGylated polystyrene plate (3881 Corning, Corning, New York), 100 µl per well. The fluorescence emission from ThT was measured at 480 nm with excitation at 448 nm using a FLUOstar Omega plate reader (BMG Labtech, Ortenberg, Germany). The plate reader was operated in quiescent mode, at a reading frequency of 0.00125 s^−1^, meaning that the only agitation applied to the samples was from the moving of the plate over the optics.

### Solubility measurements

After centrifugation in 1.5 ml Eppendorf Protein LoBind Tubes (Hamburg, Germany) of aggregated Aβ20–34 for a minimum of 30 minutes at a minimum of 14 100 RCF, 25% of the volume fraction supernatant was collected and the concentration analysed by HPLC with UV absorbance and mass spectrometry detection (Shimadzu Corporation, Kyoto, Japan). 2.0 µl was injected on a reverse phase column (BIOshell A160 Peptide CN, 5 cm × 2.1 mm, 2.7 µm). An elution gradient was used, from 5% to 95% acetonitrile (with 0.1% trifluoracetic acid) and the absorbance was measured at 256 nm. The concentration was determined from the area of the eluted peak corresponding to Aβ20–34 in comparison with a standard curve recorded for samples with known concentrations determined from the FPLC chromatogram, see above. This was confirmed by measuring the absorbance spectrum in a 10 mm quartz cuvette (Fig. S4), using a Labbot instrument (Probation Laboratories Sweden AB, Lund, Sweden). The solubility of Aβ20–34 with 10% D_2_O (pH 7.4, 20 mM NaP, 0.2 mM EDTA, 0.02% NaN_3_, 35 µM seeds) was followed using NMR spectroscopy (Fig. S3), see below for method description.

### Cryogenic transmission electron microscopy

Samples for cryo-TEM were taken at the plateau stage of the aggregation curve. Specimens for cryo-TEM were prepared in an automatic plunge freezer system (Leica EM GP). The climate chamber temperature was kept at 21 °C, and relative humidity was ≥90% to minimize loss of solution during sample preparation. 4 µL samples were loaded onto 300 mesh lacey carbon-filmed copper TEM grids and blotted with a filter paper to absorb extra solution. The grid was then plunged into liquid ethane (−180°C) to flash freeze all samples and stored in liquid nitrogen until imaging. A Fischione Model 2550 cryo transfer tomography holder was used to transfer the specimen into the electron microscope, JEM 2200FS, equipped with an in-column energy filter (Omega filter), which allows zero-loss imaging. The acceleration voltage was 200 kV and zero-loss images were recorded digitally with a TVIPS F416 camera using SerialEM under low dose conditions with a 10 eV energy-selecting slit in place.

### Small angle X-ray scattering

After 6 days of aggregation (Fig. S1), 70 µl samples were extracted from the 96 well plate and measured once with SAXS (Ganesha 300 XL) (Xenocs, Grenoble, France), using a 2D PILATUS detector (Dectris, Baden-Dättwil, Switzerland) and a GeniX Cu ULD SL X-ray source (Xenocs, Grenoble, France). The samples were all injected in the same reusable capillary (washed between samples) and incubated at 37 °C while measured at three different detector distances, covering *q*-ranges 3.77 × 10^−3^ Å^−1^ ≤ *q* ≤ 2.89 Å^−1^, 9.84 × 10^−4^ Å^−1^ ≤ *q* ≤ 7.55 × 10^−1^ Å^−1^ and 2.90 × 10^−4^ Å^−1^ ≤ *q* ≤ 2.23 × 10^−1^ Å^−1^. Buffer was also measured and subtracted from the SAXS signal.

### Microfluidic diffusional sizing

MDS (Fluidic Sciences, Royston, UK) is a technique based on diffusion and measures the ratio of labelled sample between two halves of a channel in a microfluidics device (depicted in Fig. 2). The sample with labelled protein is injected in the capillary along with flow buffer, flowing side by side in laminar flow. Over time and thereby over the length of the channel, protein will diffuse and thereby spread from one half to the other. At the end of the channel there are two chambers where fluorescence intensities of the two halves of the channel are measured. The ratio of intensities is used to calculate the average hydrodynamic size. Smaller species diffuse faster according to the Stokes–Einstein equation, giving a direct relation between diffusivity and size. Among the 5 different size ranges to choose between for each measurement, controlling the flow rate and thereby the diffusion time covered, the ranges 2–4 were used, suitable for particles between 2–17 nm. 4 µl of sample and 4 µl of buffer were used for each measurement replicate.

### Association between transient Aβ species and JB6

Purified Aβ20–34 was diluted to 6.5 mM in 20 mM NaP, 0.2 mM EDTA, 0.02% NaN_3_, pH 7.4 buffer. Samples were incubated at 37 °C in Eppendorf Protein LoBind Tubes with a PTFE coated small magnetic stirring bar (8 × 1.5 mm) (VWR, Radnor, Pennsylvania), and stirred at 1000 RPM. At different timepoints, 130 µl was removed from the sample and transferred to another 1.5 ml tube, which was centrifuged at 14 100 RCF for 30 minutes. 30 µl of supernatant was then transferred to a third tube where 10 µl of 50 nM Alexa-Fluor-488 labelled JB6 was added (final concentration of JB6, 12.5 nM). This sample was then incubated for 5 minutes before adding to an MDS plate and analyzed in terms of average hydrodynamic radius. The remaining 100 µl were thoroughly mixed and 50 µl added to a 96-well plate with 20 µM ThT and measured by single point ThT fluorescence reading.

Aβ20–34 oligomers for the binding curves with JB6 or oligomers titrated in, were prepared in the same way as described above. For the JB6 titration curve, samples were taken after 4.5 h and 5.5 h from two separate aggregation processes. After centrifugation, supernatant containing oligomers was diluted 40× resulting in final JB6 concentrations between 3–75 nM and 5.5 or 6 mM monomer (5.5 h and 4.5 h sample, respectively). By diluting with monomer instead of buffer, we attempted to keep the monomer concentration constant and prevent faster dissociation in more diluted samples. 5.5 and 6 mM monomer were estimations of the current remaining monomer concentration from the ThT intensity. All the samples with different JB6 concentration were then measured with MDS for the average hydrodynamic radius. The samples for the oligomer titration curve were taken at the broad peak between 2 h and 7 h from two separate aggregation processes. This was possible as the shape of the binding curves were very similar, meaning that the oligomer binding sites and their concentration are similar. The supernatant was then diluted from 1.33× to 7000×, also here with 5.5 or 6 mM monomer (7 h and 2 h sample respectively) and 12.5 nM JB6 in all samples. These samples were then also analysed using MDS to measure the average hydrodynamic radius.

The raw data in Fig. 4D (the fraction of fluorescent JB6 appearing in the half of the channel opposite of the inlet, *f*, as a function of volume fraction of supernatant after centrifugation, *X*) were fitted using the following eqn (1) and nonlinear least squared fitting, assuming independent binding1
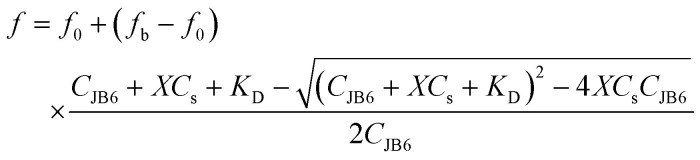
where *f*_0_ and *f*_b_ are the fractions free respective bound JB6 appearing in the half of the channel opposite of the inlet. *C*_s_ is the total concentration of JB6-binding sites in the non-diluted supernatant, *C*_JB6_ is the total JB6 concentration (fixed at 12.5 nM), and *K*_D_ is the equilibrium dissociation constant. The fitted parameters were *K*_D_, *C*_s_, *f*_0_, and *f*_b_. The value for *C*_s_ represents an upper limit to the oligomer concentration in the supernatant in the limit of one JB6-binding site per oligomer. The error analysis of *K*_D_ and *C*_s_ is presented in Section S10.

Oligomer formation of Aβ42 was measured in a similar way to Aβ20–34. Purified Aβ42, kept on ice, was diluted to 4 µM in 20 mM NaP, 0.2 mM EDTA, pH 8.0, with and without 5 µM ThT. From each solution, 100 µl was added to three wells in a Corning 96-well clear bottom half area plate (3881), and the ThT fluorescence intensity was measured continuously under quiescent conditions in a plate reader at 37 °C. An entire well without ThT was then extracted at each timepoint and centrifuged at 20 000 RCF for 5 minutes. 30 µl of supernatant was transferred to a third tube where 10 µl of 50 nM Alexa-Fluor-488-labelled JB6 was added (final concentration of JB6, 12.5 nM). This sample was incubated for 4 minutes before being added to an MDS plate and analyzed for the average hydrodynamic radius.

### Solution-state NMR spectroscopy

To determine if there is an interaction between monomeric Aβ20–34 and JB6, purified Aβ20–34 was diluted to 100 µM in 18 mM NaP, 0.18 mM EDTA, 0.018% NaN_3_, pH 7.4 buffer, 10% D_2_O, with or without 10 µM JB6. 1D ^1^H spectra were recorded with a Bruker Avance Neo 600 MHz 4-channel spectrometer with a QCI(P) 5 mm CryoProbe (Bruker Biospin, Rheinstetten, Germany). The standard pulse program zgesgppe was used. The spectral width was 16.0 ppm with 32 768 points, 1.7 s acquisition time, 256 scans, and an interscan delay of 1.0 s. The total experimental time was 12 min 7 s. TopSpin 4.3.0 was used to plot and process 1D data, using an exponential window function with line broadening of 1 Hz.

Aβ20–34 solubility was measured by transferring pooled aggregated samples with 10% D_2_O to an NMR tube, from PEGylated plates, at 4 different timepoints. 1D ^1^H spectra were recorded with a Bruker Avance Neo 800 MHz 4-channel spectrometer with a TCI 5 mm CryoProbe (Bruker Biospin, Rheinstetten, Germany). The standard pulse program zgesgppe was used. The spectral width was 15.6 ppm with 32 768 points, 1.3 s acquisition time, 128 scans, and an interscan delay of 1.0 s. The total experimental time was 5 min 14 s. TopSpin 4.3.0 was used to plot and process 1D data, using an exponential window function with line broadening of 0.3 Hz. As a standard, the same 1D ^1^H spectra were recorded of a 5 mM monomeric Aβ20–34 sample, in conjunction with each timepoint.

### Oligomer dissociation with and without JB6

Aβ20–34 samples were prepared and incubated as in the methods section for association between transient Aβ species and JB6. Here, however, 1500 µl of sample was centrifuged and 346 µl supernatant was extracted (same volume fraction as before). The supernatant was split into two tubes, one to which 58 µl of Alexa-Fluor-488 labelled 50 nM JB6 was added (final concentration 12.5 nM) and another one without JB6. The sample with added JB6 was analysed directly with MDS and then both samples were incubated at either 37 °C or 23 °C. At multiple timepoints, oligomers incubated with JB6 were analysed and from oligomers incubated without JB6, 15 µl was transferred to another tube and 5 µl of 50 nM JB6 was added (final concentration 12.5 nM). After 5 minutes incubation, this sample was also analysed.

The data in Fig. 5 are for samples taken at 2.5 h for experiments at 37 °C and from a separate aggregation process, at 4 h for experiments at 23 °C. To compare these dissociation rates, as they were extracted at different timepoints, we estimated the monomer concentration from the ThT intensity since the amount of monomer and fibril affect the net dissociation. Monomer concentrations were estimated as 5.6 and 5.4 mM at 37 °C and 23 °C, respectively (Fig. S11). These are very similar values allowing for the comparison in dissociation rate of the oligomers. Since the oligomer samples incubated with JB6 were diluted at time zero due to chaperone addition, these samples will have lower concentration of monomers, providing an increase in the net dissociation rate, compared to the oligomers incubated without JB6, which are not diluted until just before the MDS measurement. We, however, still detected stabilization against dissociation by JB6 under these experimental conditions.

## Author contributions

Conceptualization and design of the study: J. G., A. C., E. A., U. O. and S. L.; methodology: J. G., A. C., E. A., D. T., U. O. and S. L.; investigation: J. G., A. C., E. A., D. T., U. O. and S. L.; formal analysis: J. G., A. C., E. A., D. T., U. O. and S. L.; writing original draft: J. G., D. T., U. O. and S. L.; writing review and editing: J. G., A. C., E. A., D. T., U. O. and S. L.; supervision: U. O., and S. L. All authors have given approval to the final version of the manuscript.

## Conflicts of interest

There are no conflicts to declare.

## Supplementary Material

CP-028-D6CP00678G-s001

## Data Availability

Data for this article, including raw data for the main article and supplementary information (SI) are available at GitHub at https://github.com/saralinse/Published_Data/tree/PCCP_2026_Abeta_JB6_oligomer_interaction. Supplementary information is available. See DOI: https://doi.org/10.1039/d6cp00678g.
