# Supplementary material for: The chaperone DNAJB6b halts amyloid formation through association with transient Aβ oligomers
Source: Phys Chem Chem Phys. 2026 Apr 29;28(19):11897–907. doi: 10.1039/d6cp00678g (PMC13126645; doi:10.1039/d6cp00678g)
Supplement: CP-028-D6CP00678G-s001 [file CP-028-D6CP00678G-s001.pdf]

Supplementary Information (SI) for Physical Chemistry Chemical Physics.
This journal is © the Owner Societies 2026

Supplementary Information

The chaperone DNAJB6b halts amyloid formation through association with transient Aβ oligomers

Josef Getachew*[a], Andreas Carlsson[a], Emil Axell[a], Dev Thacker[a], Ulf Olsson[b], Sara Linse[a]

[a]Biochemistry and Structural Biology, Lund University, 22100 Lund, Sweden.
[b]Physical Chemistry, Lund University, 22100 Lund, Sweden.
E-mail: josef.getachew@chem.lu.se

S1. Aggregation Kinetics

Aggregation kinetics for Aβ20-34 in the presence of 0-1000 nM DNAJB6b (JB6) over the entire time-frame studied are shown in Fig. S1. There is significant inhibition at all concentrations of chaperone studied, with more pronounced inhibition the higher the JB6 concentration.

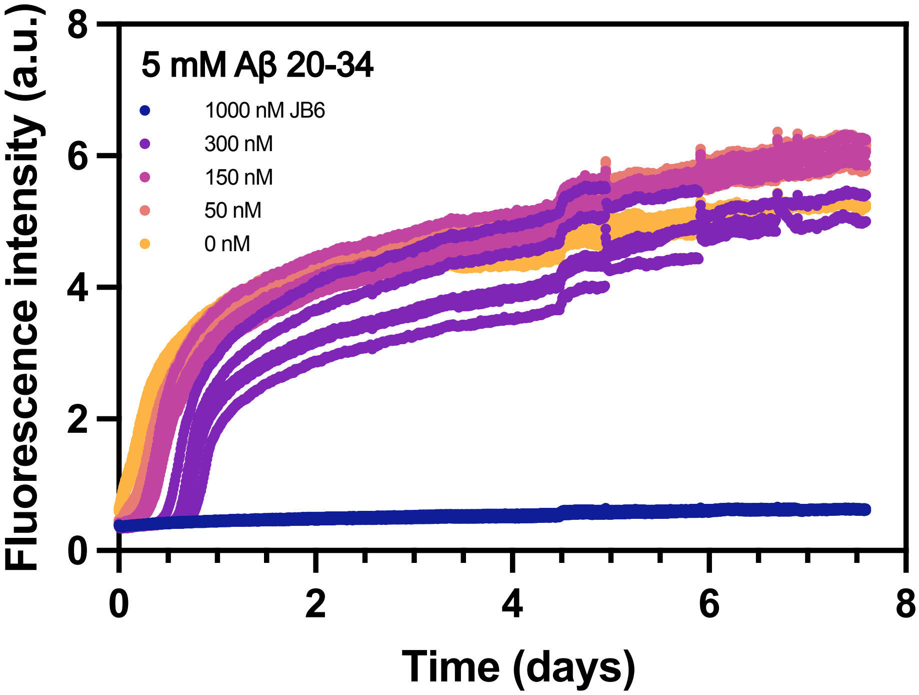

Fig. S1: Aggregation kinetics, followed by ThT fluorescence, of 5 mM Aβ20-34 with 0-1000 nM JB6 incubated at 37°C with 35 μM (0.7 %) seeds added at time zero. At time zero, N = 5 for 50-1000 nM JB6 and N = 7 for 0 nM JB6. The number of replicates decreased over the time-course, as samples were removed for characterization.

S2. Fitting Attempts to ThT Fluorescence Aβ20-34 Aggregation Kinetics

Fitting aggregation kinetics data with the master equations covering the mechanistic steps in amyloid formation may be compromised by drifts in ThT fluoresence at the aggregation plateau. This is observed in our data and its origin was therefore investigated by following Aβ20-34 aggregation at the same conditions as in Fig. 3A and S1 using ThT concentrations of 20 μM and 100 μM (Fig. S2A). There is no change in the ThT plateau drift with an increase in ThT, ruling out insufficient concentration of the dye as the explanation for the fluorescence drift. NMR spectroscopy data, section S3, also rule out an increase in aggregate formation as an explanation of the drift.

For aggregation kinetics starting in the presence of seeds, the need for primary nucleation is bypassed. Thus, to expand the dataset for fitting the rate constants, non-seeded aggregation was also conducted (Fig. S2B). Although less reproducible without seeds, fibril formation seems to be completely inhibited in presence of 100 nM JB6 at least for 115 hours; implying that inhibition of Aβ20-34 aggregation by JB6 is not only affecting secondary processes (elongation and secondary nucleation) but also primary nucleation, similar to the effect JB6 has on other amyloids [1] [2] [3].

Before fitting the kinetic traces in presence of JB6, we attempted to obtain the aggregation rate constants for Aβ20-34 alone. For this purpose, 5 mM Aβ20-34 was allowed to aggregate with varying seed concentrations (50 μM, 12.5 μM, 2.5 μM, 0.5 μM and 0 μM). The kinetic traces were fitted by three different models (Fig. S2C, D, E) (using AmyloFit [4]). The best fits were obtained using a model with primary nucleation, elongation, and secondary nucleation, in agreement with previous findings [5]. However, only the elongation rate constant, k_+ is well-determined, with a value of $1.17e^7$ $M^{-1}h^{-1}$, whereas the rate constant, k_2, and reaction order, n_2, of secondary nucleation, and the rate constant, k_n, and reaction order, n_c, of primary nucleation can vary with equally good fits. Hence, without additional data, these constants cannot be accurately obtained.

The fitted constants determined from the data in Fig. S2E were used as starting values in an attempt to explain the effect of JB6 on the rate constants k_+ and k_2. Fits to the data in Fig. 3A were done by either varying k_2 (Fig. S2F) or k_+ (Fig. S2G), keeping all other parameters globally constant. Varying k_+ gives a lower relative mean residual error (1.00) than varying k_2 (2.79), yet neither fit exhibits the increase in lag phase seen here. By varying the seed concentration, however, this behaviour could be replicated (Fig. S2H). Seemingly an inactivation of the seeds by JB6 could explain the effect up to $t_{1/2}$.

With these datasets we may interpret that JB6 has an effect on the secondary processes involved in Aβ20-34 fibril formation. However, the most accurate description of the JB6 effect would be the inactivation of seeds. Additional data would be required to more accurately discern between k_+ and k_2 and study the effect on k_n in more depth than can be concluded from Fig. S2B. However, this system is challenging to study in terms of changes in rate constants given the drift in the ThT plateau, gel formation in the aggregation plate wells, the low supersaturation of Aβ20-34 compared to other amyloid peptides, and the change in JB6 concentration during the aggregation process due to co-aggregation.

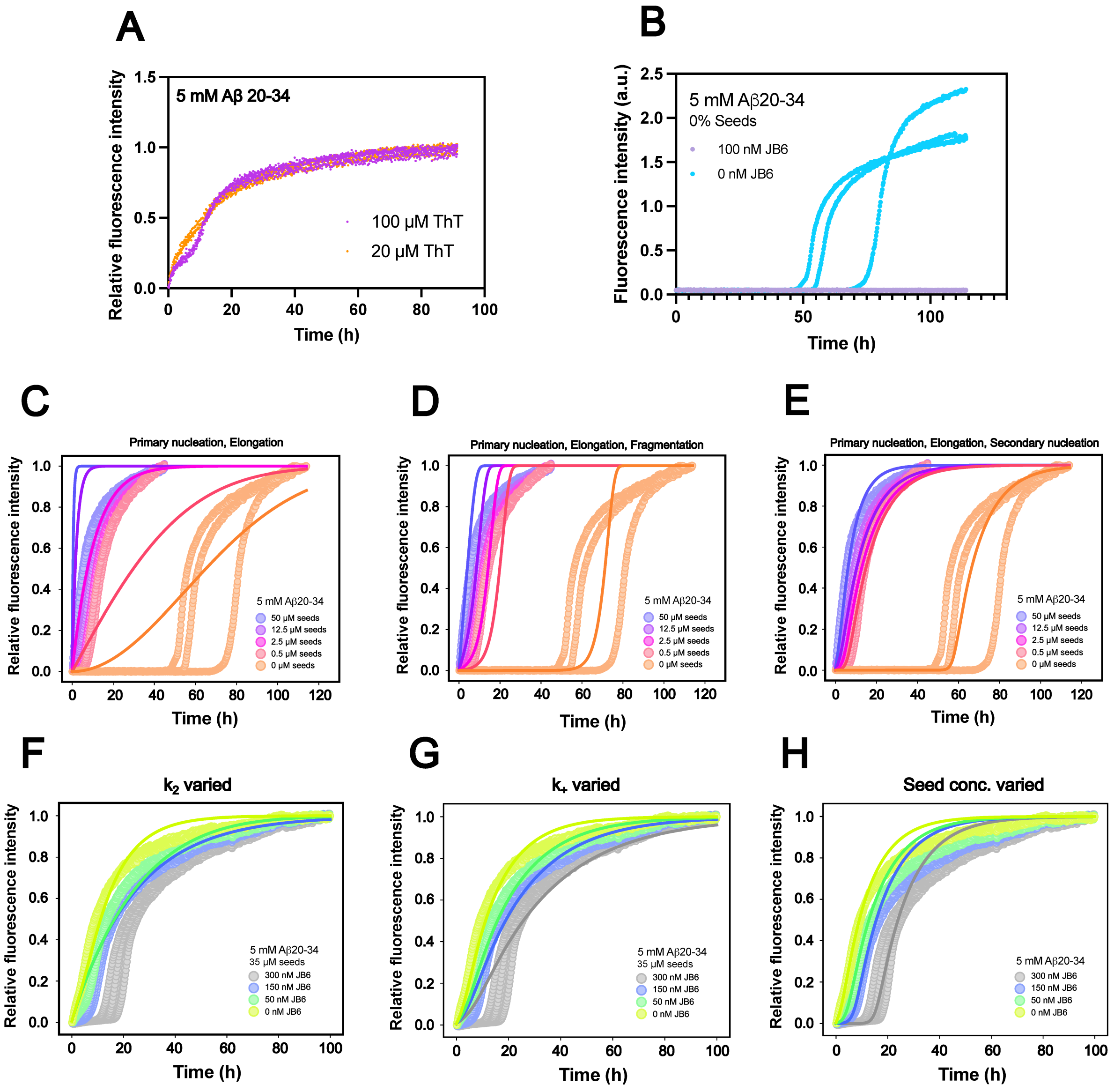

Fig. S2: Aggregation kinetics for 5 mM Aβ20-34, followed by ThT fluorescence. A) Normalized kinetic traces with 20 μM or 100 μM ThT. B) Aggregation without seeds in the presence of 100 nM or 0 nM JB6. C)-E) Fits to three different aggregation models for a dataset with varying seed concentrations (50 μM, 12.5 μM, 2.5 μM, 0.5 μM and 0 μM). C) Primary nucleation, elongation (relative mean residual error = 5.14). D) Primary nucleation, elongation, fragmentation (relative mean residual error = 2.47). E) Primary nucleation, elongation, secondary nucleation (relative mean residual error = 1.00). F)-H) Fits to secondary nucleation model with dataset in Fig. 3A. F) k_2 fitted (relative mean residual error = 2.79). G) k_+ fitted (relative mean residual error = 1.00). H) Seed concentration varied.

S3. Complementary Solubility Measurements

The peptide monomer concentration was measured by NMR as a function of aggregation time, to examine whether the drift in ThT signal at the plateau is due to further aggregation. The same samples were analysed with centrifugation and HPLC (Fig. S3A). Both methods provided highly similar concentrations. Furthermore, the concentration does not change with time after the plateau is reached, indicating that the drift in ThT signal cannot be explained by an increase in fibril mass. Possible explanations to the drift in fluorescence intensity include sedimentation of fibrils and rearrangement of ThT that lead to a higher quantum yield. Notably, we detect a higher solubility with D_2O (4.2 ± 0.1 mM), compared to without (3.5 ± 0.3 mM), as seen in Fig. S3B.

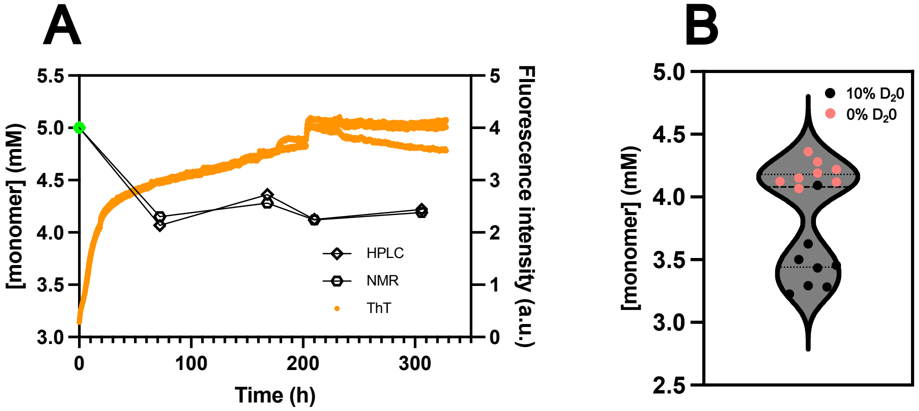

Fig. S3: Aβ20-34 solubility measurements using NMR. A) NMR and HPLC measurements of monomer concentrations during fibril formation, which is probed with ThT fluorescence. Samples withdrawn at the aggregation plateau show a constant monomer concentration, interpreted as the solubility. B) Comparing solubilities of samples with and without 10 % D_2O.

To validate the Aβ20-34 solubility value determined using HPLC, we also measured the absorbance spectrum in the far UV-range using a spectrophotometer. Aggregated Aβ20-34 was centrifuged according to the methods section in the main article and the supernatant was added to a quartz cuvette (pathlength 10 mm). Fig. S4 shows the measured absorbance spectrum, with $A_{256\ \mathrm{nm}} = 0.6831$ AU. Using the extinction coefficient of Aβ20-34, $\epsilon_{256\ \mathrm{nm}} = 197\ \mathrm{M}^{-1}\ \mathrm{cm}^{-1}$, the supernatant concentration is determined to 3.5 mM, consistent with the result obtained with HPLC.

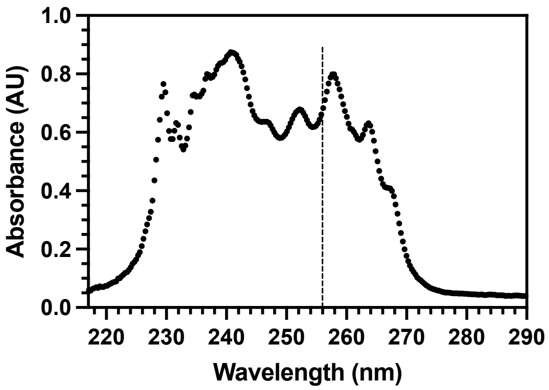

Fig. S4: Absorbance spectrum of Aβ20-34 supernatant. The dotted line highlights the wavelength 256 nm, used to quantify the peptide concentration in the supernatant.

S4. Cryo-transmission Electron Microscopy

Additional cryo-TEM images of Aβ20-34 samples taken after reaching the plateau in ThT fluorescence (Fig. S5).

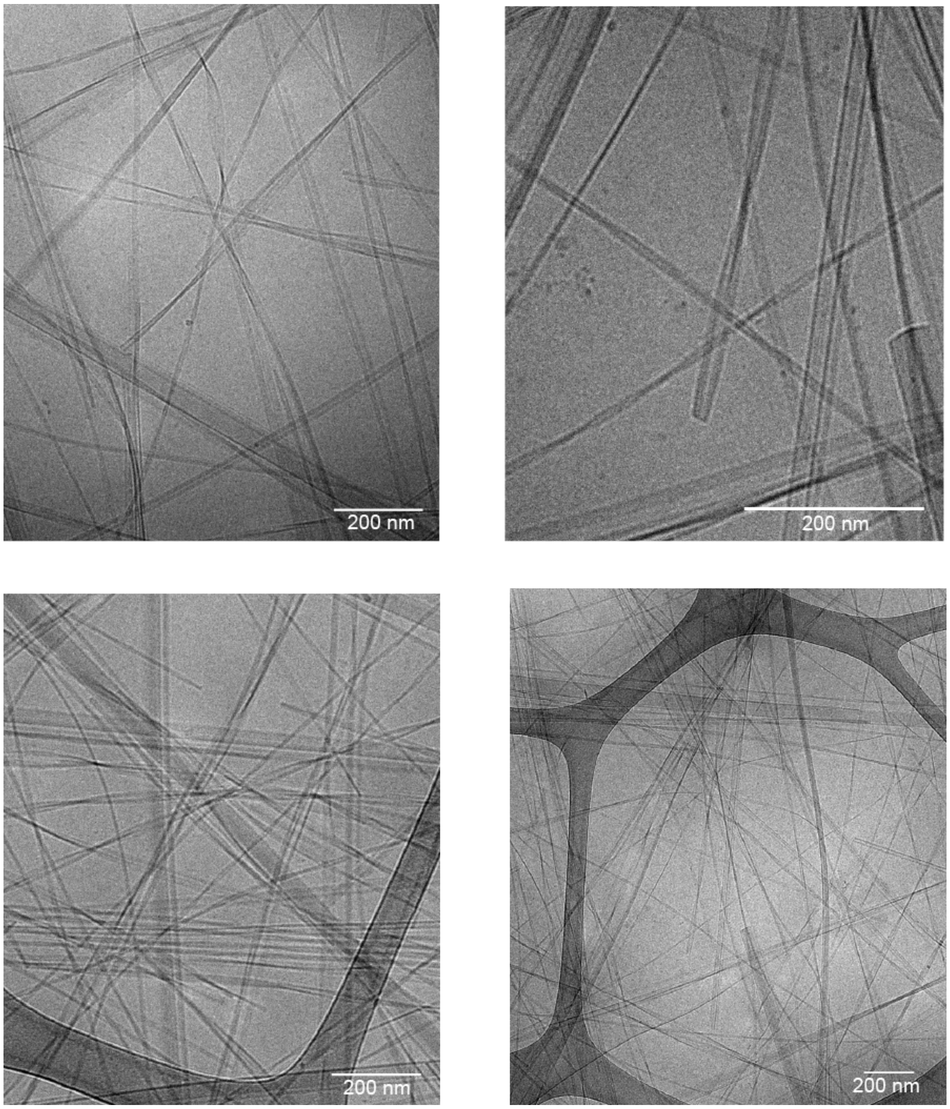

Fig. S5: Cryo-TEM images of Aβ20-34 at ThT plateau, 6 days (Fig. S1). The samples were 5-fold diluted in 20 mM sodium phosphate buffer just before freezing.

S5. Characterization of Aβ20-34 Aggregates in the Presence of JB6

SAXS patterns obtained from 5 mM Aβ20-34 aggregated in the absence (0 nM) and presence of JB6 (300 nM and 1000 nM, respectively) (Fig. S6)A. The samples were measured after 6 days of incubation (see Fig. S1). As can be seen, the scattering patterns from 0 and 300 nM JB6 essentially overlap, consistent with the same concentration of fibrils in the two samples. The sample with 1000 nM of JB6 also show a sign of fibrils at lower q-values but with approximately an order of magnitude lower intensity, and hence an order of magnitude lower fibril concentration, compared to the 0 nM sample. This implies that the aggregation in the presence of 1000 nM JB6 is still only in the initial state after 6 days of incubation. A representative cryo-TEM image of Aβ20-34 with 300 nM JB6 obtained after 6 days of incubation is presented in (Fig. S6)B. A representative cryo-TEM image of Aβ20-34 with 1000 nM JB6 obtained after 19 days of incubation is presented in (Fig. S6)C. The reason for this later time point was the slower aggregation with 1000 nM JB6. A cryo-TEM image from the sample without JB6 is shown in the main manuscript (Fig. 3D). Overall, the SAXS and cryo-TEM results show that while JB6 strongly influence the fibril formation kinetics, it has essentially no effect on the fibril structure and cross section dimensions.

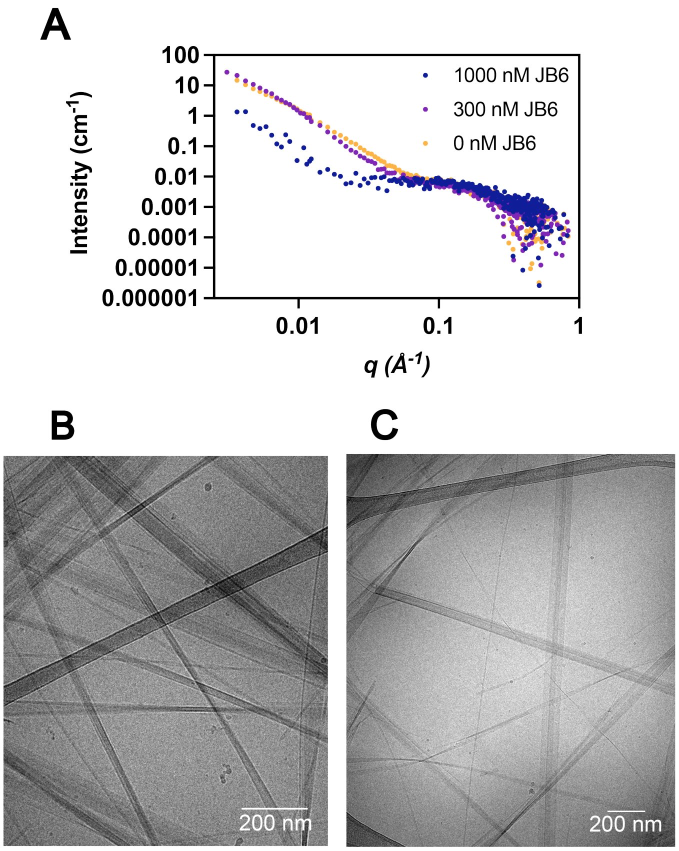

Fig. S6: Characterization of Aβ20-34 aggregates in presence of JB6 with SAXS and Cryo-TEM. **A)** SAXS pattern of samples taken at 6 days (Fig. S1). The samples with 0 and 150 nM JB6 had reached ThT plateau, whereas the 1000 nM JB6 sample remained in lag phase at this time point. **B)** Cryo-TEM image of a 5-fold diluted fibril sample in presence of 300 nM JB6, at the plateau of the aggregation process, 6 days (Fig. S1) **C)** Cryo-TEM image of a 5-fold diluted fibril sample in the presence of 1000 nM JB6, taken after aggregation (19 days).

S6. Modelling of Peptide Monomer Scattering

To analyze the contribution from peptide monomers to the SAXS pattern, the monomers were modelled as random coils. The normalized form factor, $P(q)$, is given by the Debye equation [6]

$$P(q) = 2(e^{-x} + x - 1)/(x^2) \tag{1}$$

where $x = R_g^2 q^2$, R_g being the random coil radius of gyration. The total scattered intensity, $I(q)$, depends also on the concentration, peptide molar weight and the scattering contrast and can be written as

$$I(q) = I(0)P(q) \tag{2}$$

where $I(0)$ is the forward scattered intensity at $q \to 0$. $I(0)$ can in turn be written as [7]

$$I(0) = \frac{c(\rho_p - \rho_w)^2 M_w^2}{d_m^2 N_A} \tag{3}$$

Here c is the peptide molar concentration, ρ_p and ρ_w are the scattering length densities of peptide (p) and buffer (water, w), respectively, M_w= 1491 g is the peptide molar weight, d_m is the peptide mass density and N_A=6.022·10^{23} mol^{-1} is Avogadro's number. As scattering length density values we used ρ_p=12.7·10^{10} cm^{-2}, calculated assuming a peptide mass density d_m=1.4 g/cm^3 [8] and ρ_w=9.41·10^{10} cm^{-2}, calculated assuming a water mass density of 0.997 g/cm^3. The scattering length densities were calculated using the SasView 5 software [9]. With these parameter values, the monomer concentration c was estimated from the scattered intensity.

S7. Fit of Oligomer Population

Since the MDS data in Fig. 4A reports on the average hydrodynamic radius of JB6 bound to amyloid oligomers, we interpret the radius change during the aggregation process as the relative oligomer concentration. Assuming oligomer generation is primarily stemming from secondary nucleation, the rate of oligomer formation can be described by equation 4 [10]. M(t) is the fibril mass, m(t) is the monomer mass, S(t) is the oligomer concentration, k_{o2} is the rate constant for oligomer formation via secondary nucleation, n_{o2} is its reaction order, and k_{diss} is the rate constant for oligomer dissociation.

$$\frac{dS}{dt} = k_{o2}m(t)^{n_{o2}}M(t) - k_{diss}S(t)M(t) \tag{4}$$

M(t) is fitted to a simple sigmoidal equation (equation 5), using the ThT fluorescence intensity in Fig. 4A and Fig. S7. k_f describes the slope of the sigmoidal and $t_{1/2}$ is the half-time for fibril formation. m(t) is then given from 1-M(t).

$$M(t) = \frac{1}{1 + e^{-(t-t_{1/2})*k_f}} \tag{5}$$

Equation 4 is solved numerically using MATLAB. The computed best fits are k_{o2} = 14 $M^{-0.17}h^{-1}$, n_{o2} = 0.17 (fitted with lower and upper bounds between 0-10, since n_{o2} and k_{o2} are coupled) and k_{diss} = 17 M^{-1} h^{-1}. From these fits we show that the oligomer formation in this system can be described with generation and dissociation of oligomers using existing equations applicable in similar amyloid systems.

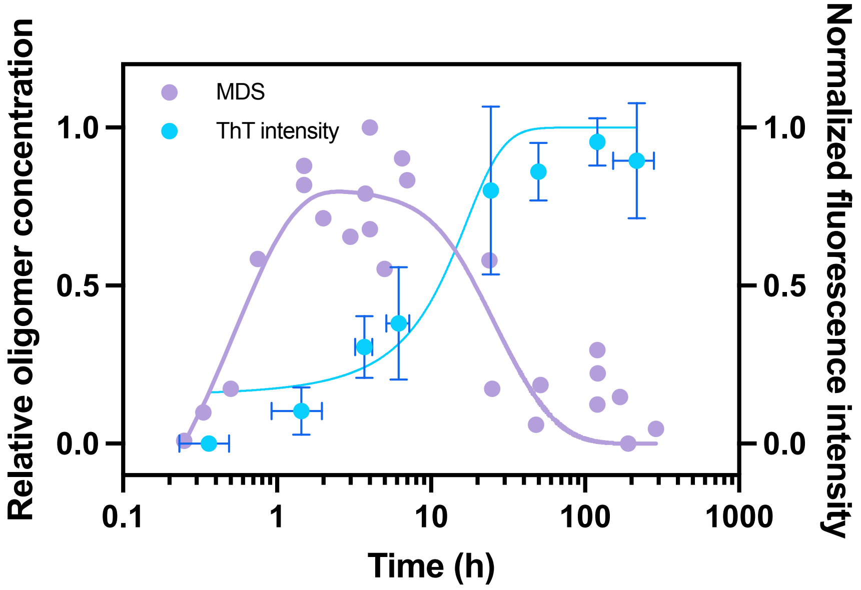

Fig. S7: Fit of oligomer and fibril formation to the data in Fig. 4A. Fibril formation is fitted to a simple sigmoidal (equation 5), seen here as the blue curve. The purple curve is the calculated oligomer population from secondary nucleation, fitted to all the individual replicates using equation 4.

S8. Cryo-TEM Images of Fibrils in Oligomeric Sample

We note that we detect fibrils in our oligomeric samples when imaging with cryo-TEM (Fig. S8). This indicates that all the fibrils in the sample are not sedimented in the current setup. We attribute this to the relatively high concentration (mM) resulting in a highly overlapping fibril network and a high viscosity. However, when mixing resuspended fibrils with JB6 and measuring with MDS, the intensity is almost completely lost and no values for $\langle R_h \rangle$ are acquired, implying that fibrils do not enter the microfluidic capillary. The intensity for the oligomeric samples in Fig. 4A, is mostly constant and significantly higher than for the pure fibrils (Fig. S9A). With the experimental timeframe for MDS measurements being shorter than the aggregation time of Aβ20-34, especially given their quiescent nature once removed from the stirring solution, these fibrils are most likely aggregates that do not sediment during centrifugation.

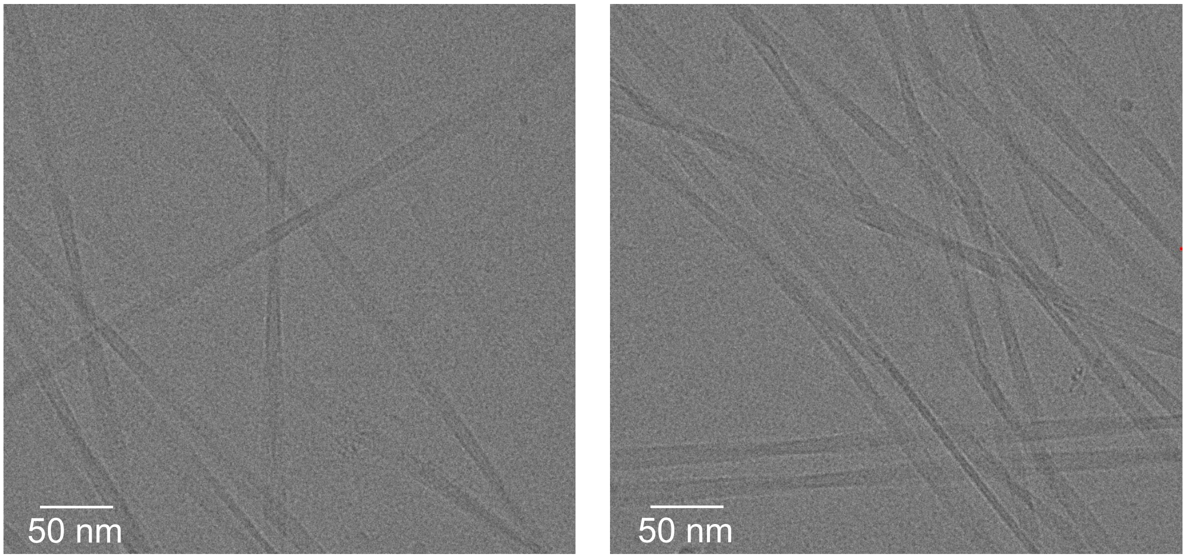

Fig. S8: Cryo-TEM images of oligomer samples showing the existence of fibrils.

S9. MDS Intensities

Intensities are measured with MDS when following oligomer interaction with JB6 or dissociation of oligomers incubated with or without JB6 (Fig. S9). The intensities are fairly constant with some increase during oligomer detection (Fig. S9A). However, when JB6 is mixed with resuspended fibrils, most of the intensity is lost. By removing all supernatant after centrifugation at 14 100 RCF for 30 minutes, and resuspending in 30 µl buffer, a sample consisting of mainly fibrils is obtained. The low intensity obtained for this sample implies that fibrils get stuck in the microfluidic chip and cannot be detected by MDS.

For the stability studies, lower intensities are measured for oligomers incubated with JB6; this could be explained as variations in the mixing of the samples (Fig. S9B). Furthermore, there is a decrease over time at 37°C. Since this is not detected at room temperature, we believe this to be a temperature effect with faster fibril formation at 37°C consuming some JB6 and decreasing the intensity. Importantly, all intensities are above the measured intensity of JB6 added to fibrils.

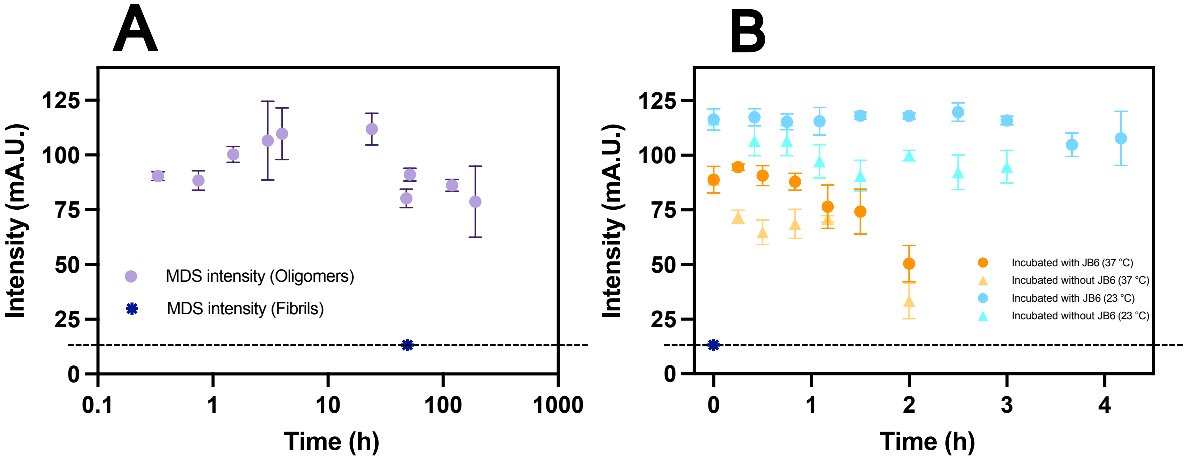

Fig. S9: Measured MDS intensities. **A)** Intensities during oligomer interaction studies in Fig. 4A. **B)** Intensities during oligomer stability studies in Fig. 5. In dark blue is the intensity of JB6 added to pure fibrils. Fibril samples are taken at 49 h. Data points are plotted as mean with error bars as STD, from N = 3 or 4 replicates.

S10. Error Analysis of Aβ20-34 Supernatant Binding Curve

Error analysis of the fit in Fig. 4D was performed by fixing K_D at a series of values while f_b, f_0 and C_s were allowed to vary. The error square sum, e.s.s., versus K_D (Fig. S10A) shows minimal change below $K_D = 1$ nM, with a $\approx$ 1.2-fold increase at K_D above 100 nM. The diffused fraction of the data in Fig. 4C were also fitted with the same equation. In this case, the e.s.s., versus K_D (Fig. S10B) does not increase below 0.1 nM and is $\approx$ 1.3-fold higher at $K_D > 100$ nM. The two independent experiments, titrating in JB6 and Aβ20-34 supernatant, respectively, show a very similar trend with a minimum below 1 nM, and an increase in the e.s.s. at increasing K_D values. Summing the e.s.s. for the K_D values included in both dataset error analyses (Fig. S10E), we observe the same behaviour with little increase in e.s.s. at $K_D < 1$ nM and a 1.25-fold increase at $K_D > 100$ nM. Therefore K_D is most likely around 1 nM or lower.

Next, we performed error analyses by fixing C_s at a series of values while f_b, f_0 and K_D were allowed to vary. For the supernatant titration, the e.s.s. is $\approx$ 7-fold higher at $C_s < 10$ nM (Fig. S10C), indicating very poor fits, with a minimum around $C_s = 1$ μM, with e.s.s. increasing slightly at higher C_s values. The JB6 titration shows an e.s.s. minimum at around 1.6 μM (Fig. S10D) with a more narrow appearance and with e.s.s. increasing $\approx$ 1.7-fold at lower C_s and $\approx$ 1.1-fold at higher C_s. Summing the e.s.s. for the C_s values included in both dataset error analyses (Fig. S10F), we observe a similar minimum around 1.5 μM.

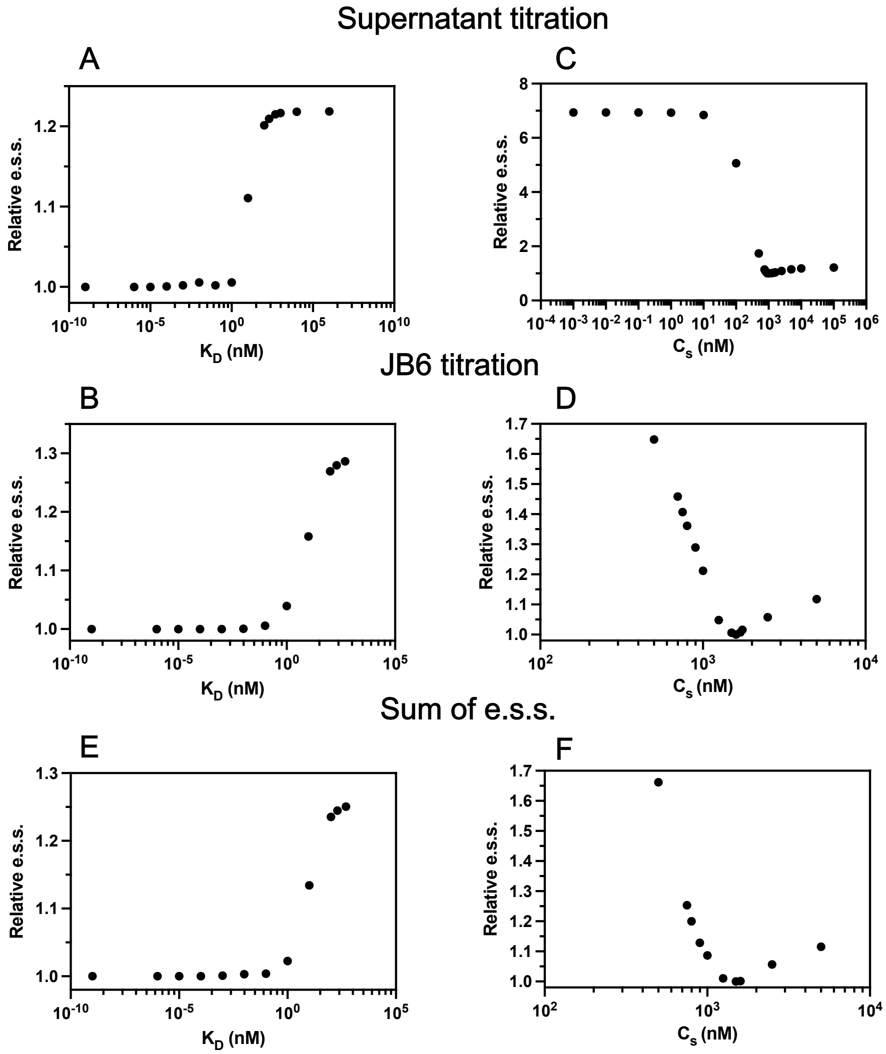

Fig. S10: Error analysis of binding curves. Relative e.s.s values (the e.s.s. divided by the e.s.s. of the best fit) for fitting to the diffused fraction data in Fig. 4C (JB6 titration) and D (supernatant titration) using fixed values of K_D or C_s, with f_0, f_b and K_D or C_s as free parameters. **A)** Error analysis of supernatant titration with varying K_D. **B)** Error analysis of JB6 titration with varying K_D. **C)** Error analysis of supernatant titration with varying C_s. **D)** Error analysis of JB6 titration with varying C_s. **E)** Sum of error analysis in A) and B) of varying K_D. **F)** Sum of error analysis in C) and D) of varying C_s.

S11. Estimation of Monomer Concentration During Oligomer Stability Studies

To compare the dissociation rate of oligomers taken at different timepoints, the monomer concentration is estimated from the ThT intensity and our solubility value of 3.5 mM ± 0.3 mM. At the highest ThT intensity, the monomer concentration is estimated as 3.5 mM, in the presence of 3 mM fibrils. From this, the monomer concentration is calculated as 5.6 and 5.4 mM at 2.5 h and 37°C and at 4 h and 23°C, respectively.

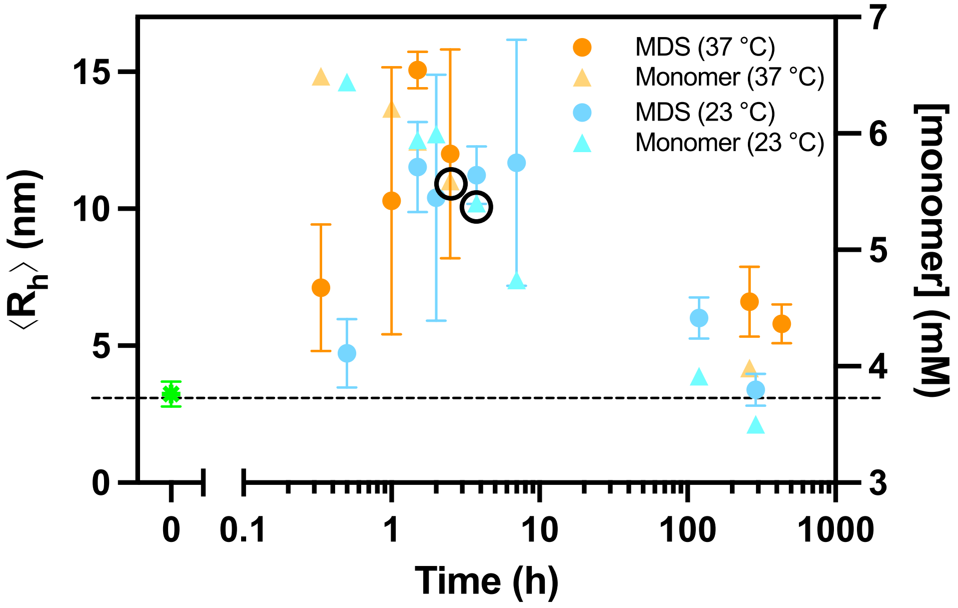

Fig. S11: Estimates of the monomer concentrations (triangles) from the ThT intensity during MDS measurements of average $\langle R_h \rangle$ (circles). Data points in MDS measurements are plotted with error bars as STD, from N = 3 or 4 replicates. In green is the $\langle R_h \rangle$ of JB6 mixed with monomeric Aβ20-34. The monomer concentrations estimated from the results of the stability studies in Fig. 5 are circled in black, as 5.6 and 5.4 mM at 37°C and 23°C, respectively.

References

[1] Cecilia Månsson, Paolo Arosio, Rasha Hussein, Harm H. Kampinga, Reem M. Hashem, Wilbert C. Boelens, Christopher M. Dobson, Tuomas P.J. Knowles, Sara Linse, and Cecilia Emanuelsson. Interaction of the molecular chaperone dnajb6 with growing amyloid-beta 42 (aβ42) aggregates leads to sub-stoichiometric inhibition of amyloid formation. *Journal of Biological Chemistry*, 289(45):31066–31076, November 2014.

[2] Cecilia Månsson, Vaishali Kakkar, Elodie Monsellier, Yannick Sourigues, Johan Härmark, Harm H. Kampinga, Ronald Melki, and Cecilia Emanuelsson. Dnajb6 is a peptide-binding chaperone which can suppress amyloid fibrillation of polyglutamine peptides at substoichiometric molar ratios. *Cell Stress and Chaperones*, 19(2):227–239, March 2014.

[3] Nicklas Österlund, Martin Lundqvist, Leopold L. Ilag, Astrid Gräslund, and Cecilia Emanuelsson. Amyloid-β oligomers are captured by the dnajb6 chaperone: Direct detection of interactions that can prevent primary nucleation. *Journal of Biological Chemistry*, 295(24):8135–8144, June 2020.

[4] Georg Meisl, Julius B Kirkegaard, Paolo Arosio, Thomas C T Michaels, Michele Vendruscolo, Christopher M Dobson, Sara Linse, and Tuomas P J Knowles. Molecular mechanisms of protein aggregation from global fitting of kinetic models. *Nature Protocols*, 11(2):252–272, January 2016.

[5] Mattias Törnquist and Sara Linse. Chiral selectivity of secondary nucleation in amyloid fibril propagation. *Angewandte Chemie International Edition*, 60(45):24008–24011, October 2021.

[6] Jan Skov Pedersen. Analysis of small-angle scattering data from colloids and polymer solutions: modeling and least-squares fitting. *Advances in Colloid and Interface Science*, 70:171–210, July 1997.

[7] O. Glatter. *Scattering methods and their application in colloid and Interface Science*. Elsevier, 2018.

[8] Hannes Fischer, Igor Polikarpov, and Aldo F. Craievich. Average protein density is a molecular-weight-dependent function. *Protein Science*, 13(10):2825–2828, October 2004.

[9] Mathieu Doucet, Jae Hie Cho, Gervaise Alina, Ziggy Attala, Jurrian Bakker, Wim Bouwman, Paul Butler, Kieran Campbell, Torin Cooper-Benun, Celine Durniak, Laura Forster, Miguel Gonzales, Richard Heenan, Andrew Jackson, Stephen King, Paul Kienzle, Jeff Krzywon, Torben Nielsen, Lewis O'Driscoll, Wojciech Potrzebowski, Stewart Prescott, Ricardo Ferraz Leal, Piotr Rozycko, Tim Snow, and Adam Washington. Sasview version 5.0.2, 2020.

[10] Catherine K. Xu, Georg Meisl, Ewa A. Andrzejewska, Georg Krainer, Alexander J. Dear, Marta Castellana-Cruz, Soma Turi, Irina A. Edu, Giorgio Vivacqua, Raphaël P. B. Jacquat, William E. Arter, Maria Grazia Spillantini, Michele Vendruscolo, Sara Linse, and Tuomas P. J. Knowles. α-synuclein oligomers form by secondary nucleation. *Nature Communications*, 15(1), August 2024.
